# Effects of Nitrogen and Phosphorus in Sediment on the Occurrence of *Cladophora* sp. (Cladophoraceae) in Aquaculture Ponds

**DOI:** 10.3390/biology13090739

**Published:** 2024-09-21

**Authors:** Yuanyuan Zhang, Kaifang Liu, Jun Lv, Xinliang Peng, Yongtao Tang, Liangjie Zhao, Yongxu Cheng, Qigen Liu

**Affiliations:** 1 Henan Academy of Fishery Sciences, Zhengzhou 450044, China; zhangyuan86123@163.com (Y.Z.); hnsclxg@163.com (J.L.); 2 College of Fisheries, Xinyang Agriculture and Forestry University, Xinyang 464000, China; liukf@xyafu.edu.cn (K.L.); pengxinliang01@126.com (X.P.); t13721071655@126.com (Y.T.); a850924t@163.com (L.Z.); 3 Centre for Research on Environmental Ecology and Fish Nutrion of the Ministry of Agriculture, Shanghai Ocean University, Shanghai 201306, China; 4 Key Laboratory of Integrated Rice-Fish Farming, Ministry of Agriculture and Rural Affairs, Shanghai Ocean University, Shanghai 201306, China

**Keywords:** nitrogen, phosphorus, sediment, *Cladophora*, benthic cyanobacteria

## Abstract

**Simple Summary:**

The overgrowth of *Cladophora* poses a threat to aquatic environments and cultured animals. This study investigated the effects of nitrogen and phosphorus levels in sediment on the growth of *Cladophora* in aquaculture ponds, which is a significant issue affecting water quality and aquatic life. We conducted microecosystem experiments to simulate varying nutrient conditions, discovering that a nitrogen-to-phosphorus ratio of 40:1 negatively impacts *Cladophora* growth. Our findings also indicated that benthic cyanobacteria, which formed dense mats on the sediment surface, can inhibit the growth of *Cladophora*, showing a strong negative correlation between their coverage and the wet weight of *Cladophora*. Furthermore, we found that total nitrogen levels in the water have a positive relationship with phytoplankton biomass, while benthic cyanobacteria coverage is linked to phytoplankton biomass through its role in phosphorus absorption and nitrogen release. Metabolite analysis identified several key compounds in benthic cyanobacteria, although none were recognized as allelochemicals or toxins. This research offers new insights into managing *Cladophora* by considering sediment nutrients and their interactions with benthic cyanobacteria, which could be valuable for improving aquaculture practices and preserving aquatic ecosystems.

**Abstract:**

To explore the impact of sedimentary nitrogen and phosphorus on *Cladophora* occurrence, we conducted a microecosystem experiment simulating different nitrogen and phosphorus content as well as nitrogen-to-phosphorus ratios in the sediment. Subsequently, to further explore the specific mechanism of influence that epiphytic algae have on *Cladophora*, we designed various microsystem culture experiments. These results revealed that an N/P ratio of 40:1 was relatively unfavorable for *Cladophora* growth. Additionally, there was an extremely significant negative correlation between the benthic cyanobacteria coverage on the sediment surface and the wet weight of *Cladophora* (*p* < 0.01), indicating that benthic cyanobacteria could inhibit the growth of *Cladophora*. Total nitrogen levels in the water column showed a significant positive correlation with phytoplankton biomass (*p* < 0.05), while benthic cyanobacteria coverage exhibited an extremely significant positive correlation with phytoplankton biomass through phosphorus absorption and nitrogen release (*p* < 0.01). Metabolite analysis of benthic cyanobacteria identified annotations for 313 metabolites; among them cis,cis-muconic acid (32.48‰), erucamide (9.52‰), phosphoric acid (6.97‰), fenpropidin (6.53‰), and propionic acid (5.16‰) accounted for proportions exceeding 5‰. However, none of these metabolites have been recognized as allelochemicals or toxins at present. This study provides novel insights into controlling *Cladophora* occurrence by considering sediment nutrients, including nitrogen and phosphorus, along with allelochemicals.

## 1. Introduction

Filamentous algae, represented by *Cladophora* and *Spirogyra*, exist widely in various natural and aquatic waters, and their overgrowth poses a threat to aquatic environments and cultured animals, through aspects such as niche occupation, nutrient depletion, and the disruption of ponds [1,2,3,4]. In aquaculture ponds, due to the shallow water depth, the overall transparency is relatively high and is essential in the early stage of aquaculture, especially for species requiring aquatic grasses such as the Chinese mitten crab, the oriental river prawn, and the red swamp crawfish. Such pond environments are thus susceptible to large-scale filamentous algal outbreaks during the low temperature season of spring. Once filamentous algae die and decompose, due to factors such as high temperature, intense light exposure, or excessive reproduction, they release toxins and emit foul odors that can lead to a deterioration in water quality and mortality among aquaculture animals [5,6,7]. To date, effective control methods for filamentous algae in aquaculture ponds have not been established. Although there are several algaecides selected from herbicides available in the market for controlling filamentous algae overgrowth [3], these nonselective herbicides also cause massive amounts of death for algae as well as aquatic grasses, which subsequently lead to significant losses among aquaculture animals [4]. In addition, some heavy metal ions, such as Cu^2+^, Zn^2+^, Pb^2+^, Cr^6+^, Ag^2+^, and Cd^2+^, have been proven to exhibit inhibitory effects on filamentous algae [8], with copper sulfate being widely employed for this purpose in aquaculture pond management. However, both herbicides and heavy metal ions pose serious threats to aquaculture production, food health, and the natural water environment.

Previous studies on the ecology of filamentous algae have presented diverse perspectives regarding the factors influencing their occurrence, including substrates [9], water flow [10], light [11], temperature [12], nutrients [13], and resting spores [14]. Interestingly in aquaculture practices, we observed that certain ponds belonging to the same farmers within identical regions exhibited similar environmental conditions and management techniques; however, some ponds experienced filamentous algae blooms while others did not. This discovery motivated us to delve deeper into the underlying causes of these blooms. Our preliminary investigation revealed consistent disparities between ponds with and without filamentous algae in terms of bioavailable nitrogen and phosphorus content ratios within sediments, as well as chlorophyll a concentrations in the water column. However, no consistent differences were observed in other parameters related to water quality, soil quality, or resting spore abundance [7]. Specifically, ponds with filamentous algae displayed higher bioavailable nitrogen and phosphorus content within their sediments compared to those without filamentous algae; conversely, ponds without filamentous algae exhibited higher bioavailable nitrogen and phosphorus ratios within their sediments along with elevated chlorophyll a concentrations in the surrounding waters. Based on these findings, we speculated that the content and ratio of bioavailable nitrogen and phosphorus in sediment might be associated with the occurrence of filamentous algae.

Considering the aforementioned reasons, we conducted a microcosm experiment manipulating nitrogen and phosphorus content and ratios in sediment. The filamentous alga chosen for this study was *Cladophora*, which exhibited the highest incidence rate in aquaculture ponds and posed significant challenges for management. The objectives of this study were twofold: (1) to investigate the suitable nitrogen and phosphorus content and ratio in sediment to inhibit *Cladophora* growth, and (2) to explore the interaction between epiphytic algae and *Cladophora*.

## 2. Materials and Methods

### 2.1. Experimental Design

In order to investigate the impact of nitrogen and phosphorus content and ratio in sediments on *Cladophora* occurrence, a microsystem experiment was designed and conducted using transparent organic glass tanks for in situ cultivation. The experiment site was located in Chongming District, Shanghai (121.5628339 E, 31.5838572 N), within a thoroughly dredged, early-stage Chinese mitten crab pond measuring 200 m^2^ in area with a depth of 50 cm. The experimental tanks had a height of 60 cm and a diameter of 21 cm. In May 2021, soil from sweet potato fields, cultivated in successive years, was collected and used as the experimental soil at the Shanghai site (available nitrogen: 2.63 mg/kg, available phosphorus: 13.44 mg/kg). This experimental soil was fully exposed, ground, and screened through a mesh size of 20. Each tank was filled with 2.0 kg of soil, resulting in a soil thickness of 5 cm. Nitrogen and phosphorus content and ratios were adjusted based on the background phosphorus content of the experimental soil using NaNO_3_ and KH_2_PO_4_ additives according to requirements listed in Table 1. The control group did not receive any additives. After thorough mixing, each group was randomly placed in the crab culturing pond where *Elodea nuttallii* grew normally. As the thickness of the soil within the experimental tanks was 5 cm, and to ensure uniformity between the inside and outside soil interfaces, the experimental tanks were pressed into the pond sediment to an approximate 5 cm depth. A total of 30 experimental tanks were set up with 3 replicates per group. The pond water, approximately 50 cm in depth, was slowly added along the sidewall of each tank. Subsequently, 10 freshly collected *Cladophora* filaments (approximately 5 cm) from the pond were introduced into each tank for experimentation purposes. Throughout this study period, daily inspections were conducted on the experimental pond to prevent potential overflow due to heavy rain or any other biological interference.

The above experiment (experiment 1) lasted for one month, and on the ninth day, epiphytic algae started to appear in some of the experimental tanks, gradually expanding their coverage areas. However, it was observed that the presence of epiphytic algae (including on the sidewall and/or the bottom) inhibited the growth of *Cladophora* to some extent. Nevertheless, several questions remained unanswered: (1) Was it the epiphytic algae on the sidewall and/or the bottom that inhibited the growth of *Cladophora*? (2) What mechanisms were involved in this inhibition? Was it due to nutrient competition, microbiota, particulate matter action, or allelopathy? To answer the above questions and elucidate the interaction between epiphytic algae and *Cladophora*, experiment 2 was designed. The specific experimental design is presented in Table 2. Experiment 2 employed a light incubator culture method with 7 experimental groups. Each experimental group had 3 replicates, with each conical bottle containing 0.30 g of wet weight of *Cladophora* filaments. The cultivation temperature was set to 26 °C with a light intensity of 4500 lx under a light-dark cycle of 12 L:12 D. The conical bottles were shaken three times daily and cultured for 5 days. It should be noted that the aforementioned culture media (filtered and unfiltered) were cultured from BG11 media under identical conditions, as mentioned above. Epiphytic algae, from both the sidewall and the bottom, were obtained from the experimental tanks, freeze-dried, and weighed before grinding.

### 2.2. Sample Collection and Analysis

After positioning the experimental tanks, water samples were collected on-site from the pond to determine water quality and quantitatively analyze phytoplankton. The in situ experiment lasted for one month without any intermediate operations. At the end of the experiment, water samples were also obtained from each tank to assess water quality and quantify phytoplankton. *Cladophora* samples were additionally collected from tanks where they grew. The coverage degree of epiphytic algae attached to the bottom was statistically estimated using a scribing method. Specifically, at the end of the experiment, the water in each tank was siphoned clean and clear photographs of their bottoms were taken from a vertical angle. Subsequently, these photos were evenly divided into multiple grids (20 × 20), and the number of grids covered by the bottom epiphytic algae was counted (a grid with less than 1 was counted as 0.5). This allowed for the estimation of both the coverage area and the degree of coverage. After collection, samples were immediately transported back to the laboratory for weighing and analysis. The wet weight of *Cladophora* was measured after removing the surface moisture using absorbent papers. Water samples were digested with alkaline potassium persulfate, and the total nitrogen content was determined through ultraviolet spectrophotometry. The total phosphorus content was determined using ammonium molybdate spectrophotometry. Quantitative phytoplankton samples were collected and immediately preserved with Lugol’s solution (30 mL/L). Species identification and counting were performed using a phytoplankton counting frame (17 mm *×* 17 mm) under an Olympus light microscope (0.196 mm^2^/field area). Each sample was randomly counted for 50 field areas after precipitation and concentration were measured for counting purposes. After counting, the average wet weights per sample for the phytoplankton were calculated according to methods published by Zhao [15].

At the end of the experiment, 0.1 g of *Cladophora* (wet weight) was cut using scissors on a cold ice bag. Subsequently, these fragments were carefully transferred into 2 mL centrifuge tubes and supplemented with 0.9 mL of PBS buffer solution (0.1 M, pH 7.4). The cells of *Cladophora* were thoroughly disrupted in an ice bath using a homogenizer, followed by centrifugation (4 °C, 2500 RPM, 10 min) to obtain the supernatant. The supernatant was then aliquoted into separate centrifuge tubes using a pipette and stored at −40 °C until analysis. Superoxide dismutase (SOD) activity, total antioxidant capacity (T-AOC), and total protein content were determined utilizing commercially available kits from the Nanjing Jiancheng Bioengineering Institute(Nanjing, China) after optimizing appropriate concentrations based on plant tissue determination methods. Additionally, the bottom epiphytic algae culture medium obtained by filtration was sent to the Shanghai Biotechnology Co., Ltd. (Shanghai, China) for metabolite analysis using LC–MS [4].

This data met the assumptions of a normal distribution and the homogeneity of variance without requiring transformations. One-way ANOVA and multiple comparisons (Duncan test) were performed using SPSS 19.0 software, with a significance level set at *p* < 0.05 to determine differences between groups. Covariance analysis was conducted to examine the effects of nitrogen and phosphorus on *Cladophora* wet weight, with *Cladophora* wet weight as the dependent variable, phosphorus content as the independent variable, and nitrogen content as the covariate. A significance level of *p* < 0.05 was used to determine statistical significance in this analysis. Pearson correlation analysis was employed using SPSS 19.0 for all correlation analyses, with a significance level set at *p* < 0.05 indicating significant correlations and *p* < 0.01 indicating highly significant correlations.

## 3. Results

### 3.1. Cladophora Growth in Experiment 1

**Figure 1:**
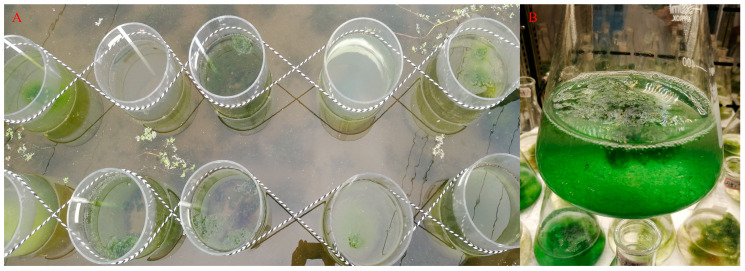
The in situ cultivation experiment in the aquaculture pond (**A**). The cultivation experiment of *Cladophora* in the light incubator (**B**).

**Figure 2:**
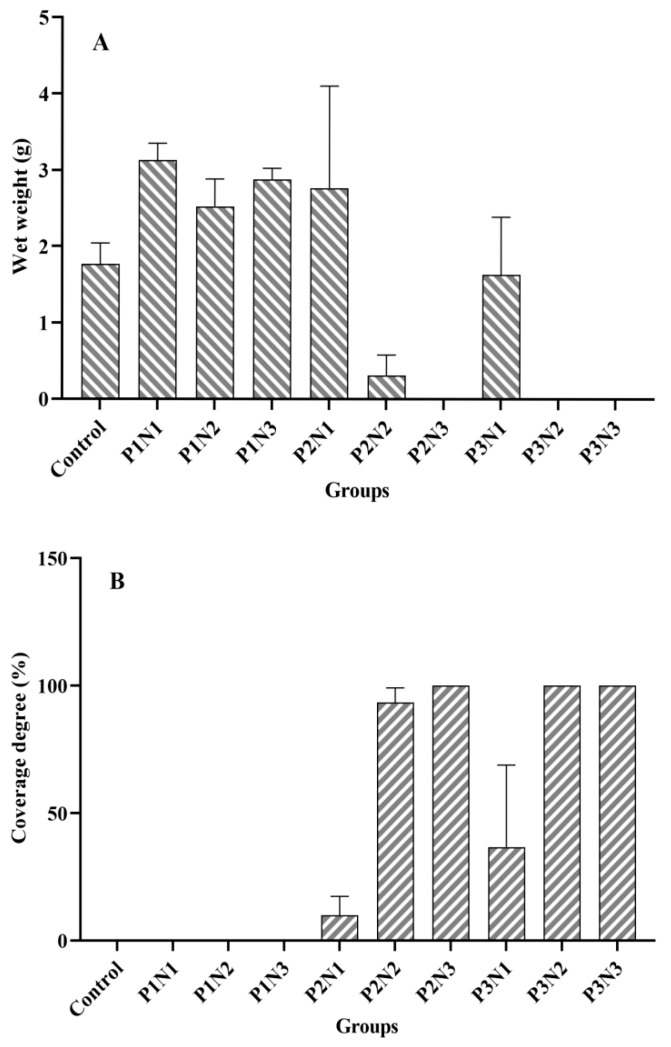
The *Cladophora* wet weight and the bottom epiphytic algae coverage degree of each experimental group. (**A**): Wet weight; (**B**): Coverage degree.

**Figure 3:**
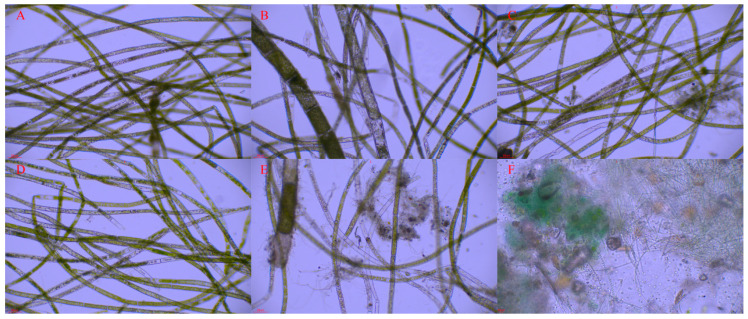
The conditions of *Cladophora* under different benthic cyanobacterial mat coverage degrees. (**A**) 0; (**B**) 30%; (**C**) 50%; (**D**) 60%; (**E**) 90%; (**F**) Photomicrograph of the benthic cyanobacterial mats.

On one hand, the content of nitrogen and phosphorus in sediments exhibited a significant effect on the wet weight of *Cladophora* at the end of the experiment (*p* = 0.027, *p* = 0.001, respectively). However, on the other hand, it was found that the experimental tanks with the bottom epiphytic algae displayed either no growth or only minimal growth of *Cladophora* with a poor developmental status (Figure 1A). The wet weight of *Cladophora* and the coverage degree of bottom epiphytic algae in each experimental group at the end of the experiment are depicted in Figure 2. This figure demonstrates a nearly symmetrical relationship between the wet weight of *Cladophora* and the coverage degree of bottom epiphytic algae. Correlation analysis revealed an extremely significant negative correlation between the wet weight of *Cladophora* and the coverage degree of the bottom epiphytic algae (r = −0.938, *p* < 0.001, n = 30). The micrographs in Figure 3A–E depict *Cladophora* under varying degrees of the bottom epiphytic algae coverage. From the figure, we found that as the degree of the bottom epiphytic algae coverage increased, the distribution of chloroplasts in *Cladophora* became progressively uneven and decreased gradually, while cell cavitations intensified gradually, resembling Figure 1B. Microscopic examination revealed that the predominant constituents of the bottom epiphytic algae were benthic cyanobacteria, including *Nostoc*, *Microcystis*, *Anabaena*, *Spirulina*, and non-heterocystous cyanobacteria. These cyanobacteria exhibited strong adhesive properties and were interconnected in a sheet-like manner (Figure 3F).

As previously mentioned, the presence of the bottom epiphytic algae significantly influenced the wet weight of *Cladophora* in the groups with P2 and P3 phosphorus levels. Therefore, an analysis of variance (ANOVA) was conducted to assess the wet weight of *Cladophora* in the four groups (Control, P1N1, P1N2, and P1N3) without the occurrence of the bottom epiphytic algae. The results revealed a significant difference in the wet weight among these four groups (*p* = 0.01). Specifically, within the P1N1, P1N2, and P1N3 groups, the wet weight of *Cladophora* was lowest in the P1N2 group and significantly lower than that in the P1N1 group (*p* = 0.022). Furthermore, all three of the aforementioned groups exhibited a significantly higher wet weight compared to the control group (*p* < 0.05).

### 3.2. The Nitrogen and Phosphorus Content and the Biomass of Phytoplankton in the Water

**Figure 4:**
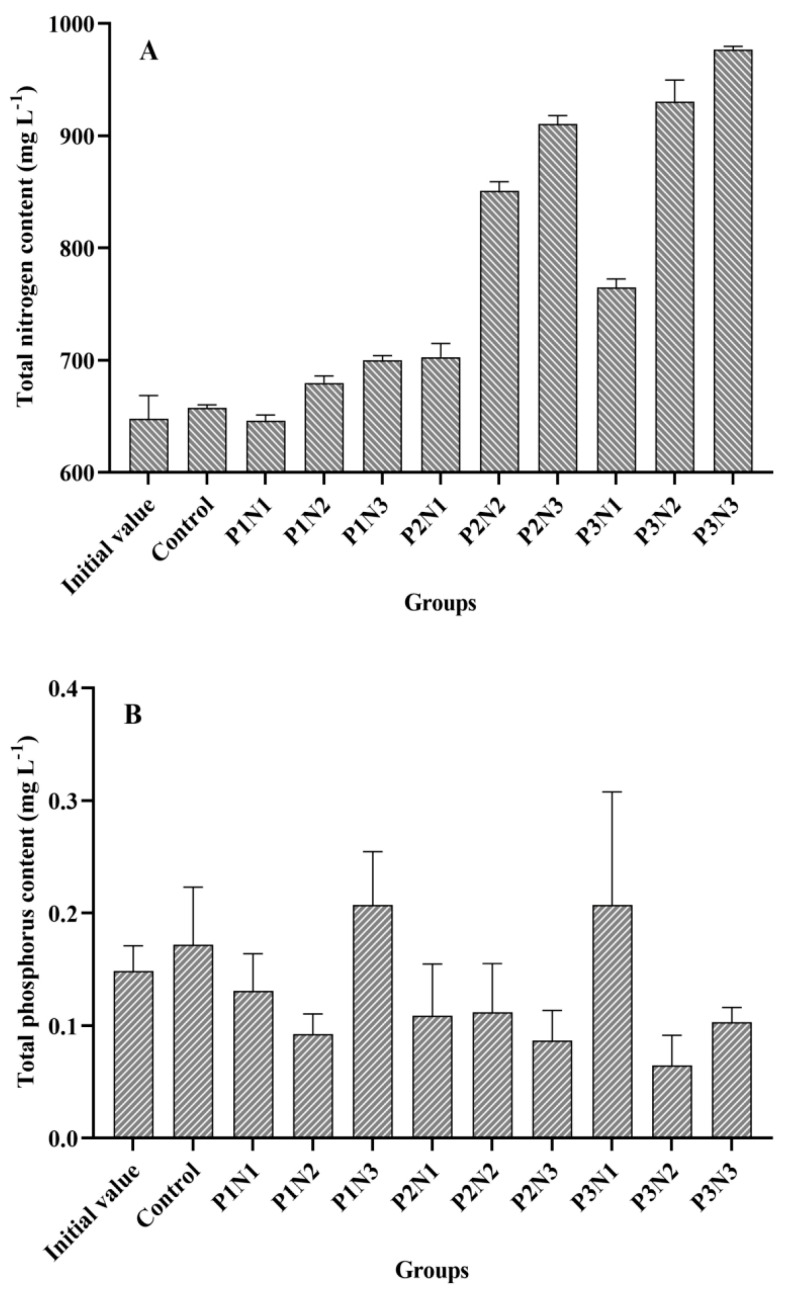
Total nitrogen and phosphorus content in the water of each experimental group. (**A**): Total nitrogen content; (**B**): Total phosphorus content.

The initial and final water TN and TP concentrations in each experimental group are presented in Figure 4. The P3N3 group exhibited the highest nitrogen concentration at 0.98 g/L, while the P3N1 group had the highest phosphorus concentration at 0.21 mg/L. Among different phosphorus concentration levels, N3 showed the highest nitrogen content, followed by N2, with N1 having the lowest content. In terms of the phosphorus concentration variation within each group, it was observed to be more volatile compared to nitrogen concentration changes. For instance, the P2N3 group displayed a lower phosphorus concentration than that of the P1N3 group; similarly, the P3N2 group had a lower phosphorus concentration than that of the P2N3 group.

**Figure 5:**
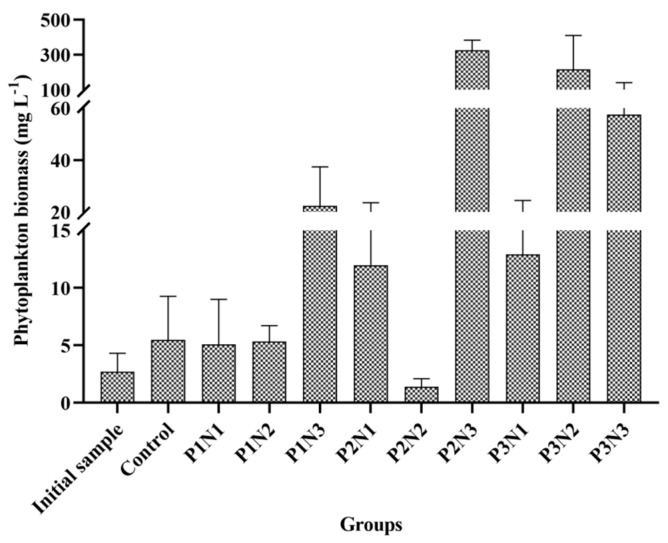
The phytoplankton biomass of each experimental group.

In this experiment, a total of 31 genera (species) were identified (Supplementary Table S1), and the biomass of phytoplankton in each subgroup is illustrated in Figure 5. Among the experimental groups, the P2N3 group exhibited the highest biomass level (0.33 g/L), followed by the P3N2 group, P3N3 group, and P1N3 group. The lowest biomass level was observed in the P2N2 group (2.71 mg/L). Comparing Figure 4 and Figure 5 revealed that phytoplankton biomass displayed a similar trend to the TN curve but an inverse trend to the TP curve. Correlation analysis demonstrated a positive association between the TN and the phytoplankton biomass (r = 0.625, n = 27), whereas TP showed a negative correlation with phytoplankton biomass (r = −0.492, n = 27); however, no significant correlations were found for these two datasets.

### 3.3. The Wet Weight Statistics of Each Experimental Group in Experiment 2

**Figure 6:**
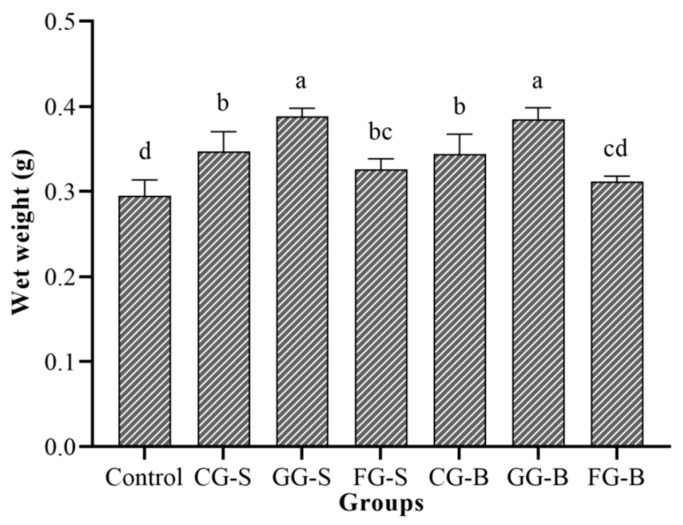
The wet weight of *Cladophora* in each experimental group. Note: Different letters indicated significant differences between groups.

After five days of cultivation in the light incubator, the wet weight of *Cladophora* in each experimental group was statistically analyzed (Figure 6). The control group exhibited a slight decrease in the wet weight by 5.2 mg, whereas the other groups demonstrated an increase. The GG-S group displayed the highest increase, with a gain of 0.0884 g, followed by the GG-B group, with an increase of 0.0847 g. The FG-B group showed the lowest increase at only 0.0115 g, and this was closely followed by the FG-S group, which had an increase of 0.0262 g. There were no significant differences observed in the wet weight between the GG-S and the GG-B groups, as well as between the CG-S and CG-B groups (*p* > 0.05). Similarly, there were no significant differences found in the wet weight between the FG-S and FG-B groups (*p* > 0.05).

### 3.4. Superoxide Dismutase Activity and the Total Antioxidant Capacity of Cladophora

**Figure 7:**
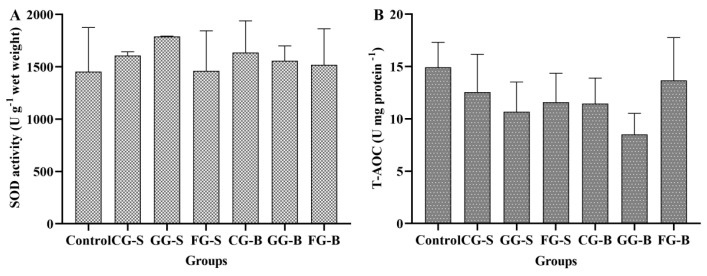
The superoxide dismutase (SOD) activity and total antioxidant capacity (T-AOC) of *Cladophora* in each experimental group. (**A**): SOD activity; (**B**): T-AOC.

To investigate the impact of different cultivation methods on enzyme activity and the antioxidant capacity of *Cladophora*, we measured the superoxide dismutase (SOD) activity and the total antioxidant capacity (T-AOC) (Figure 7). As depicted in Figure 7A, the control group exhibited the lowest SOD activity, followed by the GG-S group, and then the CG-B group. However, no significant differences were observed among these groups (*p* > 0.05). In Figure 7B, it is seen that the control group displayed the highest T-AOC level, followed by the FG-B group; conversely, both the GG-B and GG-S groups demonstrated lower levels of T-AOC. Nevertheless, there were no statistically significant differences among these groups (*p* > 0.05).

### 3.5. The Detection of Benthic Cyanobacterial Metabolites

**Figure 8:**
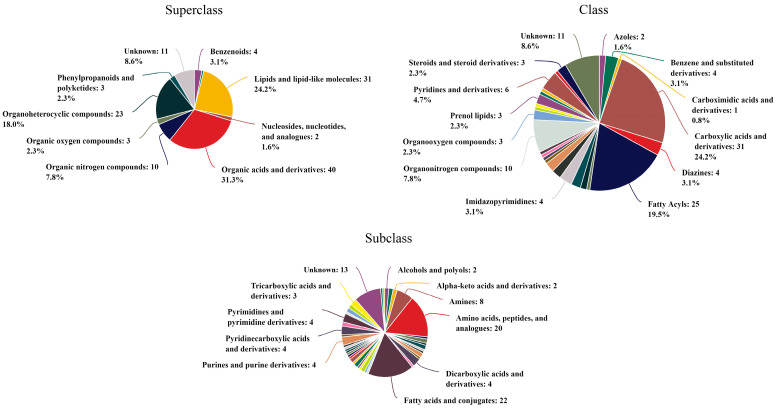
The annotated classification of metabolites at different levels.

To comprehensively investigate the impact of benthic cyanobacteria on *Cladophora*, we conducted a thorough analysis of the culture solution. A total of 10,209 metabolites were detected, out of which 313 were successfully annotated. These annotated metabolites were further categorized into different hierarchical levels (Figure 8). Among the superclass classification, organic acids and derivatives accounted for the highest proportion (31.3%), followed by lipids and lipid-like molecules (24.2%). At the class level, carboxylic acids and derivatives exhibited the highest representation (24.2%), followed by fatty acyls (19.5%). Within subclasses, fatty acids and conjugates emerged as the most abundant metabolites with a count of 22, while amino acids, peptides, and analogs ranked second with a count of 20. Notably, Table 3 presents annotations for major metabolites (>5‰), wherein cis,cis-muconic acid held the largest share at an impressive rate of 32.48‰, followed by erucamide, with a significant presence at 9.52‰.

## 4. Discussion

### 4.1. The Effect of the Nitrogen-Phosphorus Ratio in Sediment on Cladophora

The growth of *Cladophora* exhibited a non-linear correlation with the nitrogen and phosphorus content in sediment, despite both factors exerting significant effects on their growth. These findings indicated that the influence of nitrogen and phosphorus on *Cladophora* in open microecosystems is intricate and indirect, necessitating further exploration through specific data.

In the analysis of the four groups without benthic cyanobacteria, it was observed that under low phosphorus conditions, an increase in nitrogen content significantly promoted the growth of *Cladophora*. However, when the phosphorus content remained constant, the growth of *Cladophora* was also influenced by the nitrogen-phosphorus ratio. Specifically, as the nitrogen-phosphorus ratio increased, there was an overall decrease in the wet weight of *Cladophora*. Our findings indicated that an N/P ratio of 40:1 was relatively unfavorable for *Cladophora* growth in this experiment. Nevertheless, previous studies by Feng et al. [16] had found that the optimal growth condition of phytoplankton lay at an N/P ratio of 40:1 in comparison with different ratios, and its growth depended on the concentration of nitrogen mass. Therefore, it is possible that conditions with an N/P ratio of 40:1 are suitable for phytoplankton growth but inhibit *Cladophora* growth. Furthermore, Liu et al. [13] found a significant impact of N/P ratio on both *Cladophora* growth and the nutrients absorption process; they confirmed that lower N/P ratios were more conducive to *Cladophora* growth—for instance, when the N/P ratio ranged from 5–15—which resulted in higher rates of nitrogen and phosphorous absorption. This suggests that the nutrient utilization characteristics of *Cladophora* may be another factor inhibiting their growth under high N/P ratio environments.

### 4.2. Reasons for the Inhibition of Cladophora by Benthic Cyanobacteria

During the in situ cultivation process in the ponds, it was observed that both *Cladophora* and benthic cyanobacteria gradually proliferated in the experimental tanks during the initial stage. However, in the later stage, a coexistence of *Cladophora* and benthic cyanobacteria led to the gradual coverage of the bottom soil and sidewalls by the benthic cyanobacteria, while *Cladophora* exhibited shrinkage, foaming, and floating on the water’s surface. Conversely, in experimental tanks without benthic cyanobacteria, *Cladophora* were in a healthy state, and were either suspended or attached to the bottom soil or sidewalls. This phenomenon highlighted the significant influence exerted by the benthic cyanobacteria on *Cladophora*. Moreover, correlation analysis revealed an extremely significant negative correlation between the wet weight of *Cladophora* and the degree of coverage by benthic cyanobacteria (r = −0.938, *p* < 0.01, n = 30), thus confirming this impact.

Microscopic examination revealed that the benthic cyanobacteria observed in this experiment were actually benthic cyanobacterial mats composed of various benthic cyanobacteria species, such as *Oscillatoria*, *Lyngbya*, *Spirulina*, *Anabaena*, *Nostoc*, and *Phormidium* [17,18,19,20]. Among these listed species, *Nostoc*, *Anabaena*, and *Lyngbya* were found in this experiment along with *Microcystis* and *Spirulina*. These benthic cyanobacteria are capable of secreting extracellular polymeric substances (EPS) with strong adhesion properties, causing adhesion between algal cells, and between algal cells and the substrate [21]. These cohesive benthic cyanobacterial mats play important roles in nutrient absorption, adsorption, and storage, serving as reservoirs for nitrogen and phosphorus nutrients, especially phosphorus, and can absorb, store, and block the release of phosphorus from the sediment in various ways [22,23]. Consistent with these experimental findings, the water phosphorus content was lower in the experimental tanks exhibiting extensive benthic cyanobacterial coverage, while it was higher in tanks with low or no coverage of benthic cyanobacteria, despite having lower soil phosphorus content. Furthermore, it was observed that groups displaying significant growth of benthic cyanobacteria generally had relatively high phosphorus content, confirming their pronounced capacity for phosphorus absorption and utilization, as well as blocking ability. However, there were disparities between the nitrogen and phosphorus content in the water. Tanks with greater coverage of benthic cyanobacteria exhibited higher nitrogen content compared to those with lesser coverage, indicating that the emergent benthic cyanobacteria possessed a stronger affinity for absorbing or blocking phosphorus rather than nitrogen. The relationship between benthic cyanobacteria and water nitrogen content was found to be indirect, involving the interplay of benthic cyanobacterial coverage degree, the total nitrogen content, the phytoplankton biomass and the *Cladophora* biomass. Correlation analysis revealed a significant positive correlation (r = 0.655, *p* = 0.029, n = 11) between total nitrogen and the phytoplankton biomass in the water. Furthermore, there was a significantly positive correlation (r = 0.972, *p* < 0.001, n = 10) observed between the coverage degree of benthic cyanobacteria and the phytoplankton biomass. Additionally, it was confirmed that there existed a highly significant negative correlation between the wet weight of *Cladophora* and the coverage degree of benthic cyanobacteria. These findings suggested that the presence of benthic cyanobacteria exerted an inhibitory effect on *Cladophora* growth. This inhibitory effect may include two aspects: regulation of the nitrogen-phosphorus ratio via phosphorus absorption and nitrogen release by benthic cyanobacteria, as well as allelopathy effects exerted by benthic cyanobacteria on *Cladophora* growth. However, cyanobacteria constituted a relatively high proportion of the phytoplankton without being affected by allelopathy. For instance, the combined biomass of *Oscillatoria* and *Microcystis* accounted for an average of 55.18% of the total phytoplankton biomass in each group. Following the inhibitory impact of benthic cyanobacteria on *Cladophora*, phytoplankton outcompeted *Cladophora* and efficiently utilized nitrogen present in the water, resulting in an increase in its biomass with rising nitrogen content.

### 4.3. The Analysis of Benthic Cyanobacterial Metabolites

Several studies have demonstrated that cyanobacteria have a strong ability to absorb and utilize phosphorus [24,25,26]. However, their allelopathic effects on *Cladophora* remain unexplored. Nevertheless, researchers have indicated that cyanobacteria are commonly found in aquatic ecosystems and possess allelopathic properties. Their dominance in certain freshwater ecosystems following explosive growth has been attributed to the release of allelochemicals into the surrounding environment [27,28]. Notably, compounds such as fischerellin A, fischerellin B, hapalindole A, cyanobacterin, ‘cyanobacterin’ LU-1 and LU-2, nostocyclamide, and portoamides have been identified as allelochemicals produced by cyanobacteria [27,29]. Furthermore, benthic cyanobacteria exhibit a high potential for toxin production [20,30], including microcystins, cylindrospermopsins, anatoxins, saxitoxins, domoic acid, brevetoxins, geosmin, and dimethyl sulfide. Among these toxin types, those detected most frequently are microcystins and saxitoxins [31]. However, the metabolites detected in this experiment did not include the aforementioned allelochemicals or toxins. There were several possible reasons contributing to this phenomenon. On one hand, despite the well-documented allelopathic effects of numerous cyanobacteria, only a limited number of allelochemicals produced by cyanobacteria are currently identified [32,33]. On the other hand, while certain species have been reported to possess algicidal activities, such activities are typically observed within a restricted range of genera. For instance, Schlegel et al. [34] discovered that among approximately 200 cyanobacterial isolates from diverse taxonomic groups, only species from *Fischerella*, *Nostoc*, and *Microcystis* exhibited biocidal activity against green algae. Moreover, the production of toxins or allelochemicals may necessitate energy consumption; under conditions with high nitrogen and phosphorus levels, cellular energy is primarily allocated towards proliferation rather than metabolite production. Consequently, actively dividing and expanding cells are less inclined to produce secondary metabolites [35]. Additionally, some studies have also indicated that nutrient deficiency stimulates the production of toxins, such as *Nostoc* sp. [36], *Cylindrospermopsis raciborskii* [37], and *Microcystis aeruginosa* [38], which supports the aforementioned inference. Another possibility lies in that the complex mechanisms of interaction between various organisms and abiotic factors in natural environments differ significantly from those observed in laboratory cultures; thus, resulting in variations in allelochemical production [39,40]. Therefore, further research is required to investigate and verify the presence of allelochemicals within benthic cyanobacteria cultures.

## 5. Conclusions

The sedimentary nitrogen and phosphorus content exerted a significant influence on the wet weight of *Cladophora*, while the N/P ratio played an important role in their growth, with a ratio of 40/1 being relatively unfavorable. Moreover, benthic cyanobacteria on the sediment surface were found to inhibit *Cladophora* growth, exhibiting a highly significant negative correlation (*p* < 0.01). Total nitrogen in the water column was significantly positively correlated with phytoplankton biomass (*p* < 0.05), whereas the benthic cyanobacteria coverage exhibited a highly significant positive correlation with phytoplankton biomass (*p* < 0.01). The secondary metabolite detection of benthic cyanobacteria revealed the annotation of 313 metabolites, among which cis,cis-muconic acid (32.48‰), erucamide (9.52‰), phosphoric acid (6.97‰), fenpropidin (6.53‰), and propionic acid (5.16‰) accounted for proportions exceeding 5‰.

## Figures and Tables

**Table 1:** The experimental design of experiment 1.

Available Phosphorus Content	N:P	Available Nitrogen Content	Groups
P1 (13.44 mg/kg)	10:1	N1 (134.4 mg/kg)	P1N1
	40:1	N2 (537.6 mg/kg)	P1N2
	80:1	N3 (1075.2 mg/kg)	P1N3
P2 (107.52 mg/kg)	10:1	N1 (1075.2 mg/kg)	P2N1
	40:1	N2 (4300.8 mg/kg)	P2N2
	80:1	N3 (8601.6 mg/kg)	P2N3
P3 (215.04 mg/kg)	10:1	N1 (2150.4 mg/kg)	P3N1
	40:1	N2 (8601.6 mg/kg)	P3N2
	80:1	N3 (17,203.2 mg/kg)	P3N3

**Table 2:** The experimental design of experiment 2.

Attachment Location	Experimental Groups	Culture Mediums and Volume (mL)		Additives and Weight (g)
	Control group (Control)	BG11 medium (200 mL)	Ultrapure water (100 mL)	-
Sidewall	Coculture group (CG-S)	BG11 medium (200 mL)	Sidewall epiphytic algae culture medium (100 mL)	-
	Ground group (GG-S)	BG11 medium (200 mL)	Ultrapure water (100 mL)	Ground sidewall epiphytic algae (0.1 g)
	Filtration group (FG-S)	BG11 medium (200 mL)	Filtered sidewall epiphytic algae culture medium (0.22 μm) (100 mL)	-
Bottom	Coculture group (CG-B)	BG11 medium (200 mL)	Bottom epiphytic algae culture medium (100 mL)	-
	Ground group (GG-B)	BG11 medium (200 mL)	Ultrapure water (100 mL)	Ground bottom epiphytic algae (0.1 g)
	Filtration group (FG-B)	BG11 medium (200 mL)	Filtered bottom epiphytic algae culture medium (0.22 μm) (100 mL)	-

Note: The abbreviation “CG-S” stands for the experimental group “Coculture group-sidewall”. Similarly, “GG-S” stands for “Ground group-sidewall”, “FG-S” stands for “Filtration group-sidewall”, “CG-B” stands for “Coculture group-bottom”, “GG-B” stands for “Ground group-bottom”, and “FG-B” stands for “Filtration group-bottom”.

**Table 3:** The main metabolites of benthic cyanobacteria (proportion >5‰).

Metabolite	Proportion (‰)	Superclass	Class	Subclass
Cis,cis-muconic acid	32.48	Lipids and lipid-like molecules	Fatty Acyls	Fatty acids and conjugates
Erucamide	9.52	Lipids and lipid-like molecules	Fatty Acyls	Fatty amides
Phosphoric acid	6.97	Homogeneous nonmetal compounds	Nonmetal oxoanionic compounds	Nonmetal phosphates
Fenpropidin	6.53	Benzenoids	Benzene and substituted derivatives	Phenylpropanes
Propionic acid	5.16	Organic acids and derivatives	Carboxylic acids and derivatives	Carboxylic acids
Phytosphingosine	2.58	Organic nitrogen compounds	Organonitrogen compounds	Amines
Pro-Trp	1.94	Organic nitrogen compounds	Organonitrogen compounds	Amines
Citrate	1.79	Organic acids and derivatives	Carboxylic acids and derivatives	Tricarboxylic acids and derivatives
Tuberostemonine	1.69	Alkaloids and derivatives	Stemona alkaloids	Stemoamide-type alkaloids
2,4,6-trichlorophenol	1.52	Benzenoids	Phenols	Halophenols
Chenodeoxycholate	1.47	Lipids and lipid-like molecules	Steroids and steroid derivatives	Bile acids, alcohols and derivatives
Glufosinate	1.01	Organic acids and derivatives	Carboxylic acids and derivatives	Amino acids, peptides, and analogs
Palmitic acid	1.00	Lipids and lipid-like molecules	Fatty Acyls	Fatty acids and conjugates
Caffeate	0.99	Phenylpropanoids and polyketides	Cinnamic acids and derivatives	Hydroxycinnamic acids and derivatives
2-palmitoyl-rac-glycerol	0.95	Lipids and lipid-like molecules	Glycerolipids	Monoradylglycerols
3,4-dihydroxyhydrocinnamic acid	0.78	Unknown	Unknown	Unknown
Heptadecasphinganine	0.63	Organic nitrogen compounds	Organonitrogen compounds	Amines
1-stearoyl-rac-glycerol	0.57	Lipids and lipid-like molecules	Glycerolipids	Monoradylglycerols
Dl-malic acid	0.55	Organic acids and derivatives	Hydroxy acids and derivatives	Beta hydroxy acids and derivatives
Octadecanoic acid	0.54	Lipids and lipid-like molecules	Fatty Acyls	Fatty acids and conjugates
C17-sphinganine	0.51	Organic nitrogen compounds	Organonitrogen compounds	Amines

## Data Availability

Data are contained within the article.

## Supplementary Materials

The following supporting information can be downloaded at: https://www.mdpi.com/article/10.3390/biology13090739/s1, Table S1: The Genus (Species) of phytoplankton identified in each experimental group and initial water sample.
