# Supplementary material for: Effects of Nitrogen and Phosphorus in Sediment on the Occurrence of Cladophora sp. (Cladophoraceae) in Aquaculture Ponds
Source: Biology (Basel). 2024 Sep 21;13(9):739. doi: 10.3390/biology13090739 (PMC11428272; doi:10.3390/biology13090739)
Supplement: Supplementary file 1 [file biology-13-00739-s001.zip › biology-3181091-supplementary.pdf]

Supplementary table 1. The Genus (Species) of phytoplankton identified in each experimental group and initial water sample

Classification			Groups										Initial water sample
Phylum	Family	Genus (Species)	P1N1	P1N2	P1N3	P2N1	P2N2	P2N3	P3N1	P3N2	P3N3	Control	
Bacillariophyta	Naviculaceae	*Gyrosigma* sp.		+									
Bacillariophyta	Achnanthaceae	*Cocconeis* sp.	+			+			+	+	+		+
Bacillariophyta	Coscinodiscaceae	*Cyclotella* sp.		+			+	+					
Bacillariophyta	Fragilariaceae	*Synedra* sp.		+						+		+	
Bacillariophyta	Naviculaceae	*Navicula* sp.	+	+	+		+	+	+		+	+	+
Pyrrophyta	Ceratiaceae	*Ceratium* sp.		+									
Cyanophyta	Oscillatoriaceae	*Oscillatoria* sp.	+	+	+	+	+	+	+	+	+	+	+
Cyanophyta	Oscillatoriaceae	*Spirulina* sp.					+						
Cyanophyta	Merismopediaceae	*Merismopedia* sp.	+	+	+	+	+	+	+		+		
Cyanophyta	Chroococcaceae	*Chroococcus* sp.	+	+	+	+	+	+	+	+	+	+	+
Cyanophyta	Microcystaceae	*Microcystis* sp.	+	+	+	+		+	+		+	+	+
Euglenophyta	Euglenaceae	*Trachelomonas* sp.										+	
Chlorophyta	Desmidiaceae	*Euastrum* sp.		+									
Chlorophyta	Desmidiaceae	*Staurodesmus* sp.		+									
Chlorophyta	Characiaceae	*Schroederia* sp.	+	+	+	+					+	+	
Chlorophyta	Desmidiaceae	*Cosmarium* sp.			+	+			+	+	+	+	
Chlorophyta	Desmidiaceae	*Staurastrum* sp.		+									
Chlorophyta	Volvocaceae	*Eudorina* sp.	+	+	+	+							+
Chlorophyta	Oocystaceae	*Oocystis* sp.		+	+	+		+	+	+		+	+
Chlorophyta	Volvocaceae	*Pandorina* sp.			+								

Chlorophyta	Tetrasporaceae	*Tetraspora* sp.			+	+							
Chlorophyta	Chlorellaceae	*Tetraedron* sp.			+								
Chlorophyta	Chlorellaceae	*Chlorella* sp.	+	+	+	+	+	+	+	+	+	+	+
Chlorophyta	Desmidiaceae	*Staurodesmus* sp.			+								
Chlorophyta	Desmidiaceae	*Closterium* sp.	+	+	+	+	+	+	+	+	+	+	+
Chlorophyta	Chlamydomonadaceae	*Chlamydomonas* sp.		+		+	+	+		+	+	+	+
Chlorophyta	Chlorellaceae	*Selenastrum* sp.		+	+								
Chlorophyta	Scenedesmaceae	*Scenedesmus* sp.	+	+	+	+		+	+	+	+	+	+
Cryptophyta	Cryptomonadaceae	*Chroomonas* sp.										+	
Cryptophyta	Cryptomonadaceae	*Cryptomonas ovata*		+	+		+	+	+			+	+
Cryptophyta	Cryptomonadaceae	*Cryptomonas* sp.											+
